# Supplementary material for: Ultrafast infrared nano-imaging of far-from-equilibrium carrier and vibrational dynamics
Source: Nat Commun. 2022 Feb 28;13:1083. doi: 10.1038/s41467-022-28224-9 (PMC8885862; doi:10.1038/s41467-022-28224-9)
Supplement: Supplementary file 1 — Supplementary Information [file 41467_2022_28224_MOESM1_ESM.pdf]

Supporting Information for
Ultrafast infrared nano-imaging of far-from-equilibrium carrier and vibrational dynamics

Jun Nishida,[1] Samuel C. Johnson,[1] Peter T. S. Chang,[1] Dylan M. Wharton,[1] Sven A. Dönges,[1] Omar Khatib,[1] and Markus B. Raschke[1,*]

[1]*Department of Physics and JILA, University of Colorado, Boulder, CO 80309, USA*

CONTENTS

Supplementary Note 1: Experimental Setup S2

Supplementary Note 2: Theoretical Modeling S4

Supplementary Note 3: Ultrafast Nano-Imaging of Vanadium Dioxide S6

Supplementary Note 4: Ultrafast Vibrational Nano-Spectroscopy on a Lead Halide Perovskite S9

Supplementary Note 5: Comparison of Pump-Modulated and Pump-Unmodulated Detection S12

References S13

SUPPLEMENTARY NOTE 1: EXPERIMENTAL SETUP

Laser system. The output from a Yb:KGW amplified laser (Pharos, Light Conversion, 1030 nm, $\sim$1 MHz, 7 μJ) is split with $\sim$6.5 μJ pumping an optical parametric amplifier (Orpheus, Light Conversion), which generates signal and idler outputs that are then collinearly focused in a GaSe crystal to generate a mid-infrared pulse via difference frequency generation, tunable from 5–10 μm with an average pulse energy of $\sim$40 nJ. That mid-infrared pulse is attenuated to <200 pJ and is used as a probe pulse. 0.5 μJ of the amplifier output is used either at its fundamental wavelength $\lambda_{\text{pump}} = 1030$ nm or at $\lambda_{\text{pump}} = 515$ nm, which is generated by second-harmonic generation in a 2 mm-thick BBO crystal (CASTECH). The pulse duration of the $\lambda_{\text{pump}} = 1030$ nm pump is characterized to 185 fs (fwhm intensity) by SHG auto-correlation (FROG) in a 0.1 mm-thick BBO crystal (Figure S1A). The mid-infrared pulse duration of 170 fs is determined by third-order SHG-DFG cross-correlation (XFROG) between the pump and probe pulses in a 0.3 mm-thick GaSe crystal (Figure S1B).

Pump-probe implementation. The pump-probe delay is controlled by a 20-cm-long precision translation stage (SLLA42, SmarACT) with a broadband hollow retroreflector (UBBR2.5-1S, Newport) in the pump beam. After attenuation with a variable neutral density filter, the pump pulse is modulated by either a mechanical chopper (MC2000B, Thorlabs) operating at $\Omega_{\text{M}} \sim 3$ kHz ($\lambda_{\text{pump}} = 1030$ nm), or is sinusoidally modulated by an acousto-optic modulator (AOMO

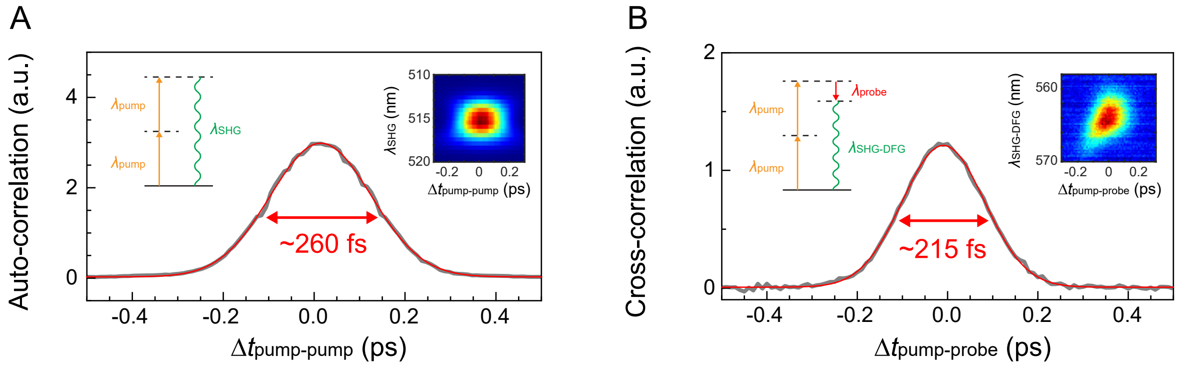

FIG. S1. **Ultrafast pulse characterization. A.** SHG intensity auto-correlation (FROG) of the 1030 nm pump pulse. **B.** Third-order SHG-DFG cross-correlation (XFROG) between the 1030 nm pump pulse and the 6000 nm probe pulse. Insets, left - nonlinear optical pathway for each signal. Insets, right - frequency-resolved optical gating (FROG) trace.

3110-120 442-633 NM, Gooch & Housego) operating at $\Omega_{\text{M}} \sim 50$ kHz ($\lambda_{\text{pump}} = 515$ nm). The modulated pump pulse is collinearly combined with the mid-infrared probe pulse and steered into an asymmetric Michelson interferometer. In the sample arm, the p-polarized pump and probe pulses are focused onto the apex of a PtIr-coated metallic tip (ARROW-NCPt, NanoAndMore USA) by an off-axis parabolic mirror (NA = 0.45). The atomic force microscope (Innova AFM, Bruker) is operated in tapping mode with a tip tapping frequency $\omega_{\text{t}} \sim 250$ kHz and a tapping amplitude $z_{\text{A}} = 60 - 70$ nm. The tip-scattered near-field signal is combined with a local oscillator from the reference arm and detected by a HgCdTe detector (KLD-0.1-J1, Kolmar Technologies). The pump pulse scattered from the tip sample region is filtered by a long-pass filter. The timing of the reference pulse is controlled by a precision translation stage (ANT95-50-L-MP, Aerotech; or SLC1760, SmarACT), which is either positioned at a certain position or continuously scanned at ~20 μm/s. The stage is programmed to generate TTL pulses every 300 nm of motion to trigger sampling by a lock-in amplifier.

Electronic signal detection. The electronic signal from the HgCdTe detector is received by a high-frequency lock-in amplifier (HF2LI, Zurich Instruments). As is established in conventional IR s-SNOM, the signal demodulated at $n\omega_{\text{t}}$ corresponds to the tip-scattered near-field signal I_{NF} dominated by the ground-state response. In contrast, the signals demodulated at $n\omega_{\text{t}} + \Omega_{\text{M}}$ and $n\omega_{\text{t}} - \Omega_{\text{M}}$ correspond to the near-field pump-probe excited-state signal ΔI_{NF} discriminated against the otherwise obfuscating ground-state response. This work uses the second-harmonic of the tip tapping frequency ($2\omega_{\text{t}}$) and its sideband modulation ($2\omega_{\text{t}} \pm \Omega_{\text{M}}$) to collect both the unpumped and the pump modulated responses.

Two-phase measurement for evaluating spectrally-averaged heterodyned near-field signal amplitude. As delineated in the main text, the spectrally-averaged amplitude relaxation $R_{\text{HPP}}(T)$ is given by the difference of recorded signals at two reference phase values $\phi = 0$ and $\phi = \pi$ for a Drude response in for, e.g., germanium or VO_2. In general, while the timing for $\phi = 0$ (constructive interference near the zero-path difference) is independent of frequency, the timing for $\phi = \pi$ (destructive interference) is technically frequency-dependent. However, the ~100 cm^{-1} fwhm bandwidth of the IR probe is effectively in the narrowband limit compared to the broad Drude response. The error in the $\phi = \pi$ delay is only ~6% (0.6 fs out of 10 fs half-cycle), which is negligible. Therefore, the two-phase method is sufficient for extracting the near-field amplitude.

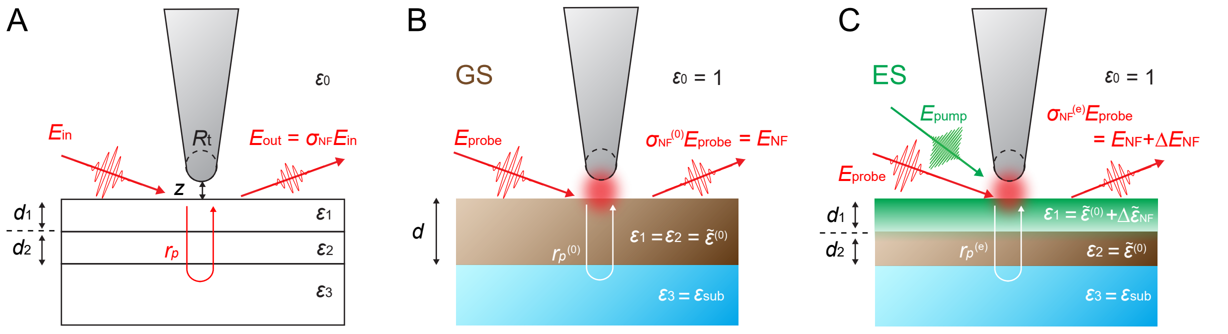

FIG. S2. **Modeling parameters. A.** Parameters defined for modeling the near-field scattering coefficient σ_{NF}, in the ground and excited states, based on the finite dipole and four-layer model. **B, C.** Parametrization for computing the ground-state (B, $\sigma_{\mathrm{NF}}^{(0)}$) and excited-state (C, $\sigma_{\mathrm{NF}}^{(\mathrm{e})}$) near-field scattering coefficients.

SUPPLEMENTARY NOTE 2: THEORETICAL MODELING

Finite dipole model with four layer model. The tip-scattered near-field signal is modeled by an established finite dipole model [1] together with a four-layer model developed for this work (Figure S2A). The configuration consists of four layers with different frequency-dependent complex valued dielectric functions $\epsilon_i(\overline{\nu})$ $(i = 0, 1, 2, 3)$. The top layer is air ($\epsilon_0 = 1$), and the bottom layer is a dielectric substrate ($\epsilon_3 = \epsilon_{\mathrm{sub}}$). The middle two layers correspond to the sample film of interest with the thickness d. In the absence of the pump pulse (Figure S2B), the middle layers (layer 1 and 2) are identical and in the ground state ($\epsilon_1 = \epsilon_2 = \tilde{\epsilon}^{(0)}$). With the pump excitation (Figure S2C), the dielectric function of the top sample layer 1 is modified to $\epsilon_1 = \tilde{\epsilon}^{(0)} + \Delta\tilde{\epsilon}_{\mathrm{NF}}$ with a thickness d_1 determined by the penetration depth of the pump pulse, while the dielectric response of the bottom sample layer 2 ($d_2 = d - d_1$) remains unmodified ($\epsilon_2 = \tilde{\epsilon}^{(0)}$). For the samples studied in this work, the penetration depth of the pump pulse exceeds the tip near-field localization of $\sim$40 nm and therefore the dielectric response in the top sample layer 1 dominates the tip-sample optical interaction. In contrast, the unperturbed sample region and the substrate only contribute to the reflection of the incident probe and of the radiated tip-scattered near-field signal.

In this configuration (Figure S2A), the near-field scattering coefficient σ_{NF} from the tip at a distance z from the sample surface is given by

$$\sigma_{\mathrm{NF}}(\overline{\nu}, z) = \alpha_{\mathrm{eff}}\{\tilde{\epsilon}_1(\overline{\nu}), z\} \cdot [1 + r_p(\overline{\nu})]^2, \tag{1}$$

with the effective polarizability $\alpha_{\text{eff}}\{\tilde{\epsilon}_1(\overline{\nu}), z\}$ described by the finite dipole model [1] and $r_p(\overline{\nu})$ the reflection coefficient for the four-layer system. The effective polarizability $\alpha_{\text{eff}}\{\tilde{\epsilon}_1(\overline{\nu}), z\}$ is computed with established formulas and tip parameters [1]. In contrast, the reflection coefficient for the p-polarized incident probe is derived from the transfer matrix method [2] and is given by

$$r_p = \frac{r_{01}e^{-i\delta_1}[e^{-i\delta_2} + r_{12}r_{23}e^{i\delta_2}] + e^{i\delta_1}[r_{12}e^{-i\delta_2} + r_{23}e^{i\delta_2}]}{e^{-i\delta_1}[e^{-i\delta_2} + r_{12}r_{23}e^{i\delta_2}] + r_{01}e^{i\delta_1}[r_{12}e^{-i\delta_2} + r_{23}e^{i\delta_2}]}, \tag{2}$$

where

$$\begin{aligned} k_i &= \frac{2\pi}{\lambda}\sqrt{\epsilon_i - \sin^2\theta_0} \\ \delta_i &= k_i d_i \\ r_{ij} &= \frac{k_i\epsilon_j/\epsilon_i - k_j}{k_i\epsilon_j/\epsilon_i + k_j}. \end{aligned} \tag{3}$$

Specific to the modeling performed here, the incident angle for the probe pulse is $\theta_0 = 60°$. $\sigma_{\text{NF}}(\overline{\nu}, z)$ is calculated for a series of sinusoidally modulated tip-sample distances z with a tapping amplitude of 60 nm. The second harmonic $\sigma_{\text{NF, 2H}}(\overline{\nu})$ is computed as the near-field scattering coefficient.

$\sigma_{\text{NF, 2H}}(\overline{\nu})$ is calculated at the ground state $\sigma^{(0)}_{\text{NF, 2H}}(\overline{\nu})$ and at the excited state $\sigma^{(\text{e})}_{\text{NF, 2H}}(\overline{\nu})$, yielding the ground-state near-field signal $E_{\text{NF}}(\overline{\nu}) = \sigma^{(0)}_{\text{NF, 2H}}(\overline{\nu})E_{\text{probe}}(\overline{\nu})$ and the excited state near-field signal $E_{\text{NF}}(\overline{\nu}) + \Delta E_{\text{NF}}(\overline{\nu}) = \sigma^{(\text{e})}_{\text{NF, 2H}}(\overline{\nu})E_{\text{probe}}(\overline{\nu})$, respectively. The difference of these two corresponds to the sideband signal detected in HPP IR s-SNOM.

Transient vibrational resonance and Fano-type interference. The simulated HPP IR s-SNOM response in Figure 2c,d (main text) is based on the sample dielectric function $\tilde{\epsilon}^{(0)}$ and its photoinduced change $\Delta\tilde{\epsilon}_{\text{NF}}$ (Figure 2b). As discussed in the main text, the narrow-band resonance in $\Delta E_{\text{NF}}(\overline{\nu})$ consists of the transient vibrational resonance and a Fano-type interference. The input dielectric function given in Figure S3A is used to assess the effect of a pure Fano-type interference. In this response, while the ground-state $\tilde{\epsilon}^{(0)}(\overline{\nu})$ exhibits a narrow-band resonance, the photoinduced $\Delta\tilde{\epsilon}_{\text{NF, car}}(\overline{\nu})$ only shows the broad Drude response. In the resulting tip-scattered near-field signals (Figure S3B), the resonance feature in $\Delta E_{\text{NF, car}}(\overline{\nu})$ is evident in the spectral vicinity of the ground-state resonance $\overline{\nu}_{\text{gs}}$. The excited state carriers drive and alter the vibrational response.

The vibrational near-field pump-probe signal $\text{Im}[\Delta E_{\text{NF, vib}}(\overline{\nu})]$ (Figure S3C) is obtained by subtracting $\text{Im}[\Delta E_{\text{NF, car}}(\overline{\nu})]$ from the total pump-probe near-field signal $\text{Im}[\Delta E_{\text{NF}}(\overline{\nu})]$ (Figure 2d).

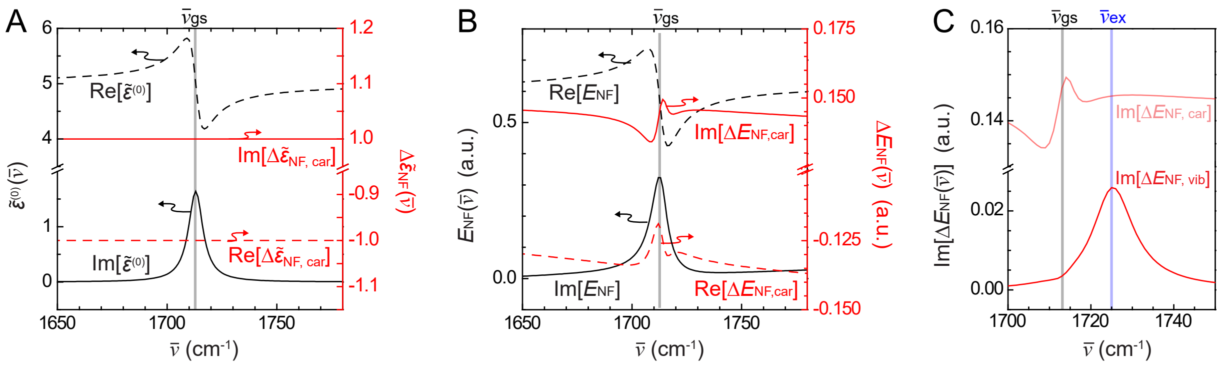

FIG. S3. **Fano-type interference induced by carrier background. A.** Model input dielectric functions identical to Figure 2b but without the transient vibrational response. **B.** $E_{\mathrm{NF}}(\bar{\nu})$ and $\Delta E_{\mathrm{NF,\,car}}(\bar{\nu})$ calculated based on the input dielectric functions, with $\Delta E_{\mathrm{NF,\,car}}(\bar{\nu})$ exhibiting Fano-type interference between the vibrational resonance and carrier background. **C.** The separated carrier and transient vibrational contributions to $\mathrm{Im}[\Delta E_{\mathrm{NF}}(\bar{\nu})]$ in Figure 2d, main text.

The spectral profile of $\mathrm{Im}[\Delta E_{\mathrm{NF,\,vib}}(\bar{\nu})]$ agrees well with the transient dielectric function $\mathrm{Im}[\Delta\tilde{\epsilon}_{\mathrm{NF}}]$ (Figure 2b), demonstrating that the transient vibrational response and Fano-type interference are approximately additive in ΔE_{NF}. In general, the separation of these two contributions can be achieved by fitting ΔE_{NF} with $\Delta\tilde{\epsilon}_{\mathrm{NF}}$. This procedure is experimetally demonstrated in Figure 5d in the main text and is further discussed below for transient vibrational nano-spectroscopy on a lead halide perovskite.

SUPPLEMENTARY NOTE 3: ULTRAFAST NANO-IMAGING OF VANADIUM DIOXIDE

Micro-Raman spectroscopy of a VO_2 nanobeam. VO_2 nanobeams are prepared by an established vapor transport method [3]. A high density of nanobeams is first prepared on a glass substrate. Then, a silicon substrate is gently pressed against the VO_2-filled surface, which results in the transfer of sparse VO_2 nanobeams to the silicon substrate. Figure S4A (solid line) shows a micro-Raman spectrum of a representative transferred nanobeam, with 532 nm excitation ($\sim$2 $\times 10^3$ W/cm^2). The observed peaks are consistent with established VO_2 phonon resonances [4] and the blue-shifted peak position of the V-O mode at $\sim$650 cm^{-1} indicates that the VO_2 nanobeams are in the monoclinic insulating M2 phase [4].

Ultrafast pump-probe IR *s*-SNOM. HPP IR *s*-SNOM is performed on a VO_2 nanowire with

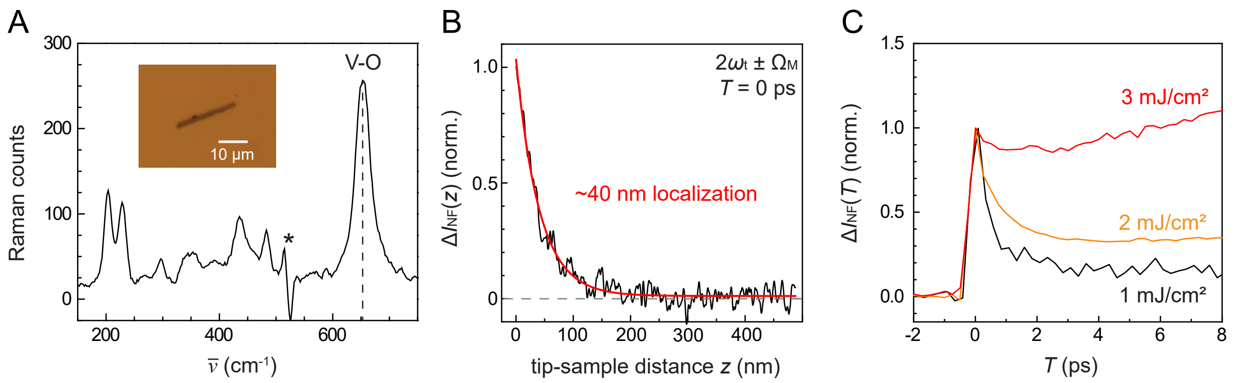

FIG. S4. **Characterization of VO_2 nanobeam.** **A.** Micro-Raman spectrum of a VO_2 nanobeam with optical image inset. $*$ - a remnant of subtracted substrate silicon Raman band. **B.** $\Delta I_{\mathrm{NF}}(z, T = 0)$ approach curve demonstrating nano-localization of the pump-probe signal. **C.** The pump fluence dependence of the VO_2 near-field pump-probe response.

a chopper modulated (3 kHz) pump (1030 nm) of varying fluence and a probe (6000 nm; $\sim$0.1 mJ/cm^2 fluence). The tip-sample distance dependence of the near-field pump-probe signal (Figure S4B) demonstrates the high degree of spatial localization ($\sim$40 nm). Figure S4C shows the power dependence of the self-homodyne near-field pump-probe signal $\Delta I_{\mathrm{SHPP}}(T)$, indicating the photoinduced IMT. At $\sim$1 mJ/cm^2, the photoinduced signal rapidly decays with only minor offset. The offset level grows as the fluence is increased and, at $\sim$3 mJ/cm^2, the slow rise in the signal level is evident, indicating the nucleation and growth of the photoinduced metallic domain, consistent with previous measurements [5–7].

Ultrafast nano-imaging of the photoinduced IMT. Ultrafast nano-imaging (40 ms pixel averaging) of the photoinduced ($\sim$2 mJ/cm^2) IMT shown in Figure 3 (main text) exhibits a clear heterogeneity along the c_{R} axis associated with local strain [8, 9], as well as a less pronounced heterogeneity across the c_{R} axis, previously attributed to non-uniform stoichiometric zoning [6]. Figure S5 shows HPP IR s-SNOM imaging performed on a VO_2 microbeam, which was previously studied in a self-homodyne pump-probe measurement [6]. The dynamical evolution of the pump-probe signal $R_{\mathrm{HPP}}(T)$ exhibits more pronounced heterogeneity across the c_{R} axis. Thus, the two mechanisms, inducing transient heterogeneity along and across the c_{R} axis, appear to generally co-exist in VO_2, depending on the morphology of each nanobeam.

On the potential optical origin of the observed heterogeneity. The low conductivity of photoinduced carriers in an only partially metallic VO_2 nanobeam excludes a potential transient

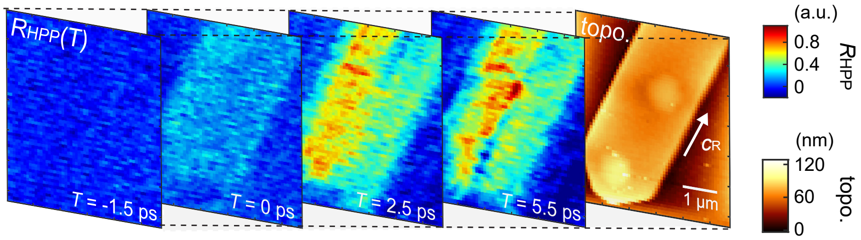

FIG. S5. **Photoinduced IMT in a VO_2 microbeam.** In contrast to the data shown in Figure 3, this microbeam exhibits the photoinduced heterogeneity transverse with respect to the c_R axis.

optical antenna effect, where a nanobeam acts as a mid-infrared resonator and exhibits an enhanced response at its terminals.

The spatial non-uniformity in the pump excitation could also lead to the observed heterogeneity in the near-field pump-probe signal $R_{HPP}(T)$. However, the excitation of the nanobeam is likely homogeneous since the pump beam size at the focus ($\sim$10 μm) is much larger than the dimensions of the nanobeam. Figures S6A-B show the topography of two similarly oriented nanobeams to the rod shown in Figure 3d in the main text, where the c_R axis is nearly parallel to the propagation direction of the incident pump. The nanobeams are also comparable in their heights ($\sim$300 nm), leading to similar excitation profiles among the three nanobeams and yet exhibit distinct spatial profiles in the near-field pump-probe signals. This demonstrates that the observed spatial heterogeneity in the near-field pump-probe signals is an inherent property of each nanobeam and cannot be attributed to the excitation profile of the pump beam. Further, Figure S6C shows a nanobeam oriented perpendicular to the incident beam direction and shows a strong enhancement at the edges. The key bulk behavior of the heterogeneity both along and across the c_R axis is evident.

These data sets together demonstrate that purely optical effects, such as the antenna effect and non-uniform excitation profile, do not account for the observed heterogeneity in the near-field pump-probe signals.

Retrieval of the dielectric function. For the retrieval of $\Delta\tilde{\epsilon}_{NF}$ through model fitting, the VO_2 ground-state dielectric constant $\tilde{\epsilon}^{(0)} = 6.6$ at the probe wavelength of 6 μm is taken from a previous far-field study [10]. The corresponding substrate (silicon) dielectric constant is $\epsilon_{sub} = 11.7$. Since the absorption in VO_2 is weak, the visible pump penetrates the entire thickness of the nanobeam ($d_1 = 300$ nm and $d_2 = 0$ nm in Figure S2). To find $\Delta\tilde{\epsilon}_{NF}$, its value is varied until it simultaneously fits the ground- (E_{NF}) and excited-state (ΔE_{NF}) responses, yielding $\Delta\tilde{\epsilon}_{NF} \sim 0.25+1.0i$ in our case.

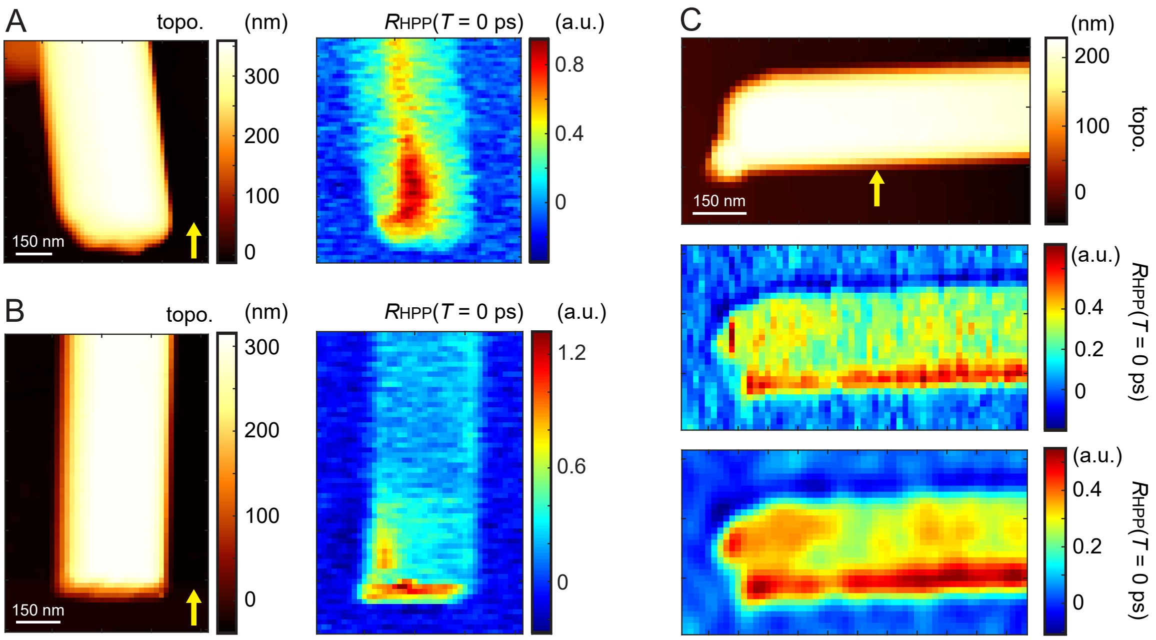

FIG. S6. **Assessment of optical effects in photoinduced heterogeneity. A, B.** Topography (left) and heterodyned near-field pump-probe signal profiles (right) of two nanobeams which are oriented similarly to the rod in Figure 3. Yellow arrows in the topography images show the direction of the incident pump beam. Despite similar orientations, the three rods exhibit distinct spatial profiles in the near-field pump-probe signals. **C.** Top: topography of a nanobeam that is oriented perpendicular to the other rods. Middle: corresponding near-field pump-probe image. Bottom: cubic splined image of the middle image with enhanced contrast, demonstrating the transient domains both parallel and perpendicular to c_R axis, as also observed in other rods.

SUPPLEMENTARY NOTE 4: ULTRAFAST VIBRATIONAL NANO-SPECTROSCOPY ON A LEAD HALIDE PEROVSKITE

Triple cation perovskite FAMACs. The triple cation perovskite sample studied in this work is a mixture of three different A-site cations of formamidinium (FA^+), methylammonium (MA^+) and cesium (Cs^+). The perovskite has an overall composition of $[(FA_{0.83}MA_{0.17})_{0.95}Cs_{0.05}]Pb(I_{0.83}Br_{0.17})_3$ and is prepared by a solution-processed spin-coating method [11]. A ~600 nm thick film is coated on a glass substrate for nano-spectroscopy and on a CaF_2 window for conventional far-field pump-probe spectroscopy.

Far-field transmission vibrational spectroscopy. For conventional far-field pump-probe

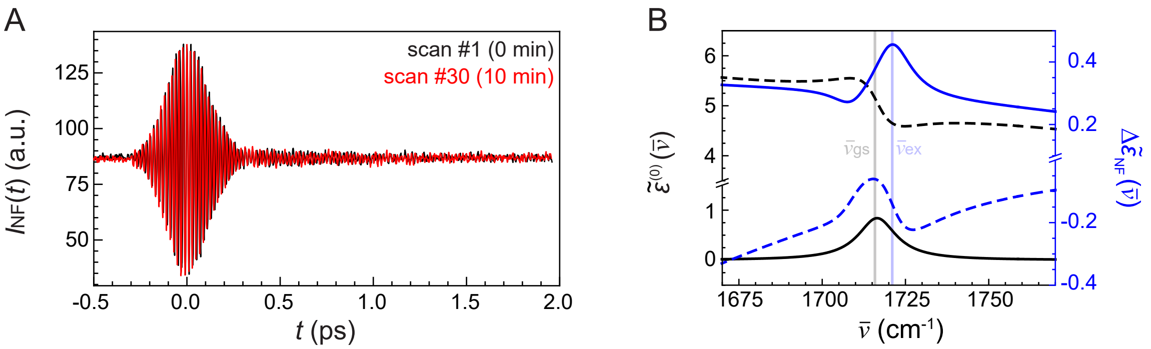

FIG. S7. **Transient vibrational nano-spectroscopy of a lead halide perovskite. A.** Single I_{NF} interferograms demodulated at $2\omega_{\mathrm{t}}$ at the beginning and end of 10 minutes of data collection, acquired under constant illumination of the visible pump pulse with pump-probe timing delay $T = 2$ ps. Both interferograms are not normalized or offset, indicating no sign of sample photo-degradation. **B.** Retrieved nano-localized ground (black) and pump-induced (blue) complex dielectric functions from $E_{\mathrm{NF}}(\bar{\nu})$ and $\Delta E_{\mathrm{NF}}(\bar{\nu})$ in Figure 5c based on model fitting.

spectroscopy (Figure 4c), the collinearly propagating pump (515 nm) and probe (5830 nm) beams are focused with a $f = 10$ cm silver-coated off-axis parabolic (OAP) mirror onto the sample surface. The transmitted IR probe is collected and collimated by an identical OAP mirror and is directed into a symmetric Michelson interferometer for spectral characterization. The probe interferogram is detected with a HgCdTe detector and lock-in amplified. The photoinduced change ΔT is demodulated at the pump modulation frequency $\Omega_{\mathrm{M}} \sim 10$ kHz. With the pump fluence of $\sim$50 μJ/cm^2 and pump-probe time delay of 0.5 ps, the acquired signal level is $\Delta T/T \sim 0.5\%$.

Transient vibrational nano-spectroscopy: experiment. The pump (515 nm) and probe (5830 nm) beams are collinearly focused to the s-SNOM tip apex with identical fluence ($\sim$250 μJ/cm^2). The AFM and sample compartment are purged with nitrogen gas ($<1\%$ O_2) to suppress photoinduced degradation. A 15 μm $\times$ 10 μm domain is selected to characterize the heterogeneity of polaron-cation coupling. Measurement locations are separated from each other by > 5 μm beyond the pump focus size so that prolonged illumination at one location does not influence subsequent measurements. At each location, conventional heterodyne (at $2\omega_{\mathrm{t}}$) and sideband nanoscale pump-probe heterodyne (at $2\omega_{\mathrm{t}} \pm \Omega_{\mathrm{M}}$) interferograms are collected at the pump-probe time delay of $T = 2$ ps by scanning the interferometer stage at 20 μm/s (spectral resolution $\sim$ 15 cm^{-1}). Over 30 interferograms are collected in $\sim$10 min for averaging. Figure S7A demonstrates measurement stability by comparing an interferogram at the beginning and end of a scan at the same location.

Transient vibrational nano-spectroscopy: analysis. The sideband interferogram ΔI_{NF} is subtracted from the conventional interferogram I_{NF}, yielding the pure ground-state response. The ground-state and the pump-probe interferograms are averaged, Fourier transformed, referenced to a non-resonant response (germanium), and deconvolved with the $E_{\text{LO}}(\overline{\nu})$ to yield the ground-state and pump-probe spectra $E_{\text{NF}}(\overline{\nu})$ and $\Delta E_{\text{NF}}(\overline{\nu})$. The ground-state spectral phase is baselined so that $E_{\text{NF}}(\overline{\nu})$ is purely real aside from the resonance arising from the CN anti-symmetric stretch mode of the FA cation [12]. Identical phase baselining parameters are applied to $\Delta E_{\text{NF}}(\overline{\nu})$, allowing for the phase-locked comparison of $\Delta E_{\text{NF}}(\overline{\nu})$ to $E_{\text{NF}}(\overline{\nu})$, which is critical for fitting.

Retrieval of the transient dielectric function. The non-resonant ground-state dielectric response of the perovskite ($\tilde{\epsilon}_{\infty}^{(0)} = 5$), pump penetration depth (100 nm; based on the absorption coefficient of the perovskite film characterized by UV-Vis absorption spectroscopy), and substrate dielectric constant ($\epsilon_{\text{sub}} \sim 1.69$) are assumed. $\Delta\epsilon_{\text{NF}}(\overline{\nu})$ is varied with a broadband response (quadratic) and narrowband resonances (Lorentzians for each vibration) to fit ΔE_{NF}. Figure S7B shows the retrieved dielectric function $\Delta\epsilon_{\text{NF}}(\overline{\nu})$ from fitting the data in Figure 5c.

Polaron-cation coupling constant. We attribute the observed blue shift in the molecular vibrational peak position to its coupling to the polaron absorption, which is reported to be observed around 1200 cm^{-1} [13]. We describe the coupling between the polaron absorption and molecular vibration with a simple Hamiltonian of

$$\hat{H} = \begin{pmatrix} \tilde{\nu}_{\text{pol.}} & J \\ J & \tilde{\nu}_{\text{vib.}} \end{pmatrix}, \tag{4}$$

where $\tilde{\nu}_{\text{pol.}} \sim 1200$ cm^{-1} and $\tilde{\nu}_{\text{vib.}} = 1715$ cm^{-1} are the uncoupled polaron absorption and the molecular vibrational energies, respectively, and J is the coupling constant. Diagonalizing the Hamiltonian gives

$$\tilde{\nu}_{\pm} = \frac{\tilde{\nu}_{\text{pol.}} + \tilde{\nu}_{\text{vib.}} \pm \sqrt{4J^2 + (\tilde{\nu}_{\text{pol.}} - \tilde{\nu}_{\text{vib.}})^2}}{2}. \tag{5}$$

$\tilde{\nu}_{+}$ corresponds to the transient vibrational frequency. Figure S8 plots the transient peak shift $\Delta = \tilde{\nu}_{+} - \tilde{\nu}_{\text{vib.}}$ with respect to the varying coupling constant J. The observed peak shift of 5 – 8 cm^{-1} in the near-field pump-probe measurement corresponds to a polaron-cation coupling constant of 50 – 70 cm^{-1}.

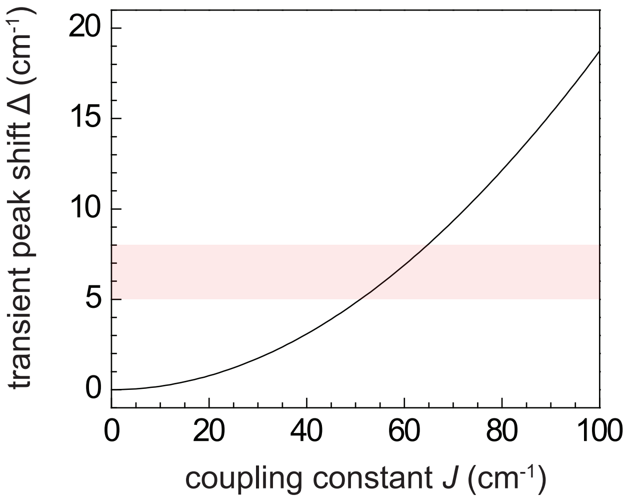

FIG. S8. **Relationship between polaron-cation coupling constant and transient vibrational peak shift.** The shift in the vibrational peak position observed in the excited state, induced by the coupling between the polaron absorption and molecular vibration as described in Eq. (5) for various coupling constant J.

SUPPLEMENTARY NOTE 5: COMPARISON OF PUMP-MODULATED AND PUMP-UNMODULATED DETECTION

Figure S9A compares data acquired by conventional pump-unmodulated detection and our pump-modulated detection, showing the T-dependent near-field pump-probe amplitude $\Delta I_{\mathrm{NF}}(T)$ of a VO_2 nanobeam. The pump-probe relaxation acquired in a conventional manner (demodulated at $2\omega_{\mathrm{t}}$) exhibits low-frequency noise which obscures the plateau level reached after an initial fast relaxation. However, the sideband demodulated signal ($2\omega_{\mathrm{t}} \pm \Omega_{\mathrm{M}}$) filters out the slow noise and the plateau level is quantitatively resolved.

Figure S9B shows the spectrally resolved near-field pump-probe signal $\mathrm{Im}[\Delta E_{\mathrm{NF}}]$ of a lead halide perovskite at $T = 2$ ps. The unmodulated pump-probe spectrum, collected at $2\omega_{\mathrm{t}}$, is dominated by noise, while the pump-modulated spectrum, collected at $2\omega_{\mathrm{t}} \pm \Omega_{\mathrm{M}}$, clearly shows the excited-state transient vibrational response.

As demonstrated in these two examples, particularly with low duty cycle excitation, excitation modulation and sideband lock-in detection are critical for the sensitive and quantitative evaluation of near-field pump-probe signals. In the main text, we provide the signal-to-noise enhancement factor of >4 for the sideband demodulated signal at $2\omega_t \pm \Omega_{\mathrm{M}}$ in comparison to the pump-probe

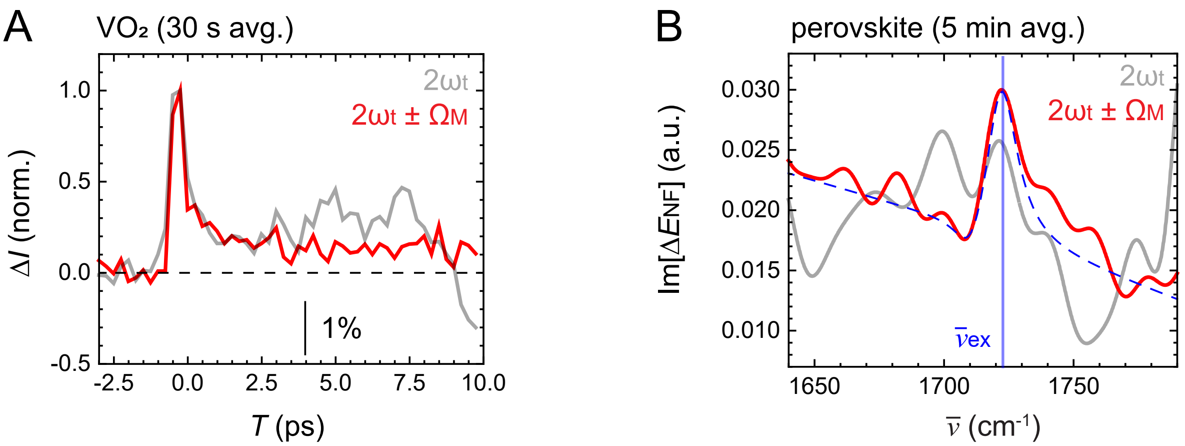

FIG. S9. **Comparison of the excitation modulated and unmodulated detection. A.** The T-dependent near-field pump-probe signal ΔI_{NF} demodulated at $2\omega_t$ (pump unmodulated) and $2\omega_t \pm \Omega_{\mathrm{M}}$ (pump-modulated) channels. **B.** The spectrally resolved near-field pump-probe signal observed at $T = 2$ ps. While the $2\omega_t$ channel is dominated by noise, in $2\omega_t \pm \Omega_{\mathrm{M}}$ channel the peak associated with the transient vibrational resonance is evident.

signal observed at $2\omega_t$. This is estimated by comparing the standard deviations of the data points for the two data sets in Fig. S9A after $T = 4$ ps, where the signal variation is dominated by the noise. Each data point in Fig. S9A is acquired in 110 ms, yielding a full pump-probe profile in 30 seconds.

* markus.raschke@colorado.edu

[1] Govyadinov, A. A., Amenabar, I., Huth, F., Carney, P. S. & Hillenbrand, R. Quantitative Measurement of Local Infrared Absorption and Dielectric Function with Tip-Enhanced Near-Field Microscopy. *J. Phys. Chem. Lett.* **4**, 1526–1531 (2013).

[2] Katsidis, C. C. & Siapkas, D. I. General transfer-matrix method for optical multilayer systems with coherent, partially coherent, and incoherent interference. *Appl. Opt.* **41**, 3978 (2002).

[3] Wu, J. *et al.* Strain-induced self organization of metal-insulator domains in single-crystalline VO_2 nanobeams. *Nano Lett.* **6**, 2313–2317 (2006).

[4] Atkin, J. M. *et al.* Strain and temperature dependence of the insulating phases of VO_2 near the metal-insulator transition. *Phys. Rev. B* **85**, 020101 (2012).

[5] Cocker, T. L. *et al.* Phase diagram of the ultrafast photoinduced insulator-metal transition in vanadium dioxide. *Phys. Rev. B - Condens. Matter Mater. Phys.* **85**, 155120 (2012).

[6] Dönges, S. A. *et al.* Ultrafast Nanoimaging of the Photoinduced Phase Transition Dynamics in VO_2. *Nano Lett.* **16**, 3029–3035 (2016).

[7] O'Callahan, B. T. *et al.* Inhomogeneity of the ultrafast insulator-to-metal transition dynamics of VO_2. *Nat. Commun.* **6**, 1–8 (2015). 1412.5495.

[8] Huber, M. A. *et al.* Ultrafast Mid-Infrared Nanoscopy of Strained Vanadium Dioxide Nanobeams. *Nano Lett.* **16**, 1421–1427 (2016).

[9] Jones, A. C., Berweger, S., Wei, J., Cobden, D. & Raschke, M. B. Nano-optical investigations of the metal-insulator phase behavior of individual VO_2 microcrystals. *Nano Lett.* **10**, 1574–1581 (2010).

[10] Wan, C. *et al.* On the Optical Properties of Thin-Film Vanadium Dioxide from the Visible to the Far Infrared. *Ann. Phys.* **531**, 1900188 (2019).

[11] Nishida, J., Alfaifi, A. H., Gray, T. P., Shaheen, S. E. & Raschke, M. B. Heterogeneous Cation–Lattice Interaction and Dynamics in Triple-Cation Perovskites Revealed by Infrared Vibrational Nanoscopy. *ACS Energy Lett.* **5**, 1636–1643 (2020).

[12] Glaser, T. *et al.* Infrared Spectroscopic Study of Vibrational Modes in Methylammonium Lead Halide Perovskites. *J. Phys. Chem. Lett.* **6**, 2913–2918 (2015).

[13] Munson, K. T., Kennehan, E. R., Doucette, G. S. & Asbury, J. B. Dynamic Disorder Dominates Delocalization, Transport, and Recombination in Halide Perovskites. *Chem* **4**, 2826–2843 (2018).
